# Temperature Dependence of the Electrical Properties of Na_2_Ti_3_O_7_/Na_2_Ti_6_O_13_/POMA Composites

**DOI:** 10.3390/molecules27185756

**Published:** 2022-09-06

**Authors:** Salomão Dos Santos Costa, Juliana Pereira da Silva, Matheus Moraes Biondo, Edgar Aparecido Sanches, Marcos Marques Da Silva Paula, Francisco Xavier Nobre, José Anglada Rivera, Yohandys Alexis Zulueta, Milton S. Torikachvili, David Vieira Sampaio, Marcos Vinicius Dias Vermelho, Ştefan Ţălu, Lianet Aguilera Dominguez, Yurimiler Leyet

**Affiliations:** 1 Department of Physics, Federal University of Amazonas, Manaus 69067-005, AM, Brazil; 2 Department of Materials Engineering, Federal University of Amazonas, Manaus 69067-005, AM, Brazil; 3 Federal Institute of Education, Science and Technology of Amazonas, Manaus 69020-120, AM, Brazil; 4 Department of Physics, Universidad de Oriente, Santiago de Cuba 90500, Cuba; 5 Department of Physics, San Diego State University, San Diego, CA 92182-1233, USA; 6 Institute of Physics, Federal University of Alagoas, Maceió 57072-900, AL, Brazil; 7 The Directorate of Research, Development and Innovation Management (DMCDI), Technical University of Cluj-Napoca, 400020 Cluj-Napoca, Romania; 8 Technological Development Institute (INDT), Manaus 69075-351, AM, Brazil

**Keywords:** ceramic/polymer composites, electrical characterization, electrical resistivity

## Abstract

The temperature dependence of the electrical properties of composites formed by biphasic sodium titanate and poly(*o*-methoxyaniline) (Na_2_Ti_3_O_7_/Na_2_Ti_6_O_13_/POMA) with different concentrations of POMA (0%, 1%, 10%, 15%, 35% and 50%) in the ceramic matrix was determined from measurements of complex impedance. The structural details were studied by means of X-ray diffraction, confirming the formation of the Na_2_Ti_3_O_7_/Na_2_Ti_6_O_13_/POMA composites. The displacement of the (200) reflection from 2θ = 10.45° to 11.15° in the composites with 10 and 15% of POMA suggested the partial replacement of H^+^ for Na^+^ in the Na_2_Ti_3_O_7_ structure. The thermal properties were investigated by Thermogravimetry and Differential Thermal Analysis. The Thermogravimetry curves of the composites with POMA content of 1, 10 and 15% presented profiles similar to that of pure sodium titanate sample. The composites with 35 and 50% of POMA showed a process at temperatures around 60–70 °C, which was associated with water absorbed by the polymer. The analysis of the complex impedance spectroscopy measurements revealed that the electrical resistivity of the composites in the range from 0 to 35% increased by two orders of magnitude, with different values for each concentration. This positive temperature coefficient of resistivity was less noticeable in the composite with highest POMA mass content (50%). The rapid increase in resistivity caused an increase in the relaxation time calculated from the time domain. The electrical response of the 50% of POMA compound changes in relation to what was observed in the other compounds, which suggests that there is a saturation limit in the increase in resistivity with POMA content.

## 1. Introduction

Ceramic-polymer composites have been a promising alternative for the electronics industry due to their ability to control ionic conductivity and mechanical strength [1,2,3]. Typical inorganic components have been admixed to polymeric-based composites including silicon oxide (SiO_2_) [4], titanium oxide (TiO_2_) [5], aluminum oxide (Al_2_O_3_) [6], Li_10_GeP_2_S_12_ (LGPS) [7], Li_7_La_3_Zr_2_O_12_ (LLZO) [8], Li_1.5_Al_l0.5_Ge_1.5_P_3_O_12_ (LAGP) [9], and Li_6.4_La_3_Zr1._4_Ta_0.6_O_12_ (LLZTO) [10]. In what pertains to polymers, composites containing polyethylene oxide (PEO) continue to be widely studied; however, the use of conducting polymers such as polyaniline (PANI) and polypyrrole (PPy) can also be highlighted [11,12,13]. Displaying structural and physicochemical properties similar to PANI, poly(*o*-methoxyaniline) (POMA) is an “Intrinsically Conducting Polymer” (ICP) employed in several technological applications, including electromagnetic shielding in the microwave range [14], manufacture of films for electroluminescent devices [15], as well as in hybrid and composite films for ammonia gas sensors (NH_3_) [16,17]. Moreover, POMA can also be found in nanocomposites for applications in electrochemical supercapacitors [18], dye-sensitized solar-cell electrodes [19], composites containing carbon nanotubes or graphene [20], and deoxyribonucleic acid (DNA) for biosensors [21]. This interest in the technological application is motivated by POMA’s interesting properties, such as biocompatibility, cost-effectiveness, ease of synthesis, low density, high mechanical strength, electrical conductivity, as well as the possibility of fine-tuning structural modifications [14,20].

Sodium titanates are versatile ceramics presenting excellent ion exchange capability, allowing their application in photocatalytic processes [22], sensors [23], capacitors [24,25], sodium ion battery electrodes, lithium ion [26,27,28] and solid state electrolytes [29].

A recent report on ceramic-polymer composite (CPC) containing sodium titanates (Na_2_Ti_3_O_7_/Na_2_Ti_6_O_13_) and POMA [30] revealed that the addition of the polymer drastically improved the electrical behavior of the ceramic matrix. The composite containing 50% (*w/w*) POMA presented an electrical resistivity of 8.18 × 10^4^ Ω·cm at room temperature (T), about 3 orders of magnitude lower than the value of pure sodium titanate (1.00 × 10^7^ Ω·cm).

The temperature dependence of the electrical resistivity of the sodium titanates in the range of 25–250 °C was recently reported [31]. The two-phase Na_2_Ti_3_O_7_/Na_2_Ti_6_O_13_ displayed a positive coefficient of resistivity as a function of temperature (PTCR) near room temperature. The resistivity increased by nearly three orders of magnitude from 25 °C to 70 °C, it peaks around 75 °C, and started to drop linearly as the temperature was raised further to 250 °C. In contrast, measurements of ρ(T) for POMA thin films showed a decrease of two orders of magnitude from 20 °C to 110 °C [32]. Despite the opposite temperature dependences of ρ(T) for Na_2_Ti_3_O_7_/Na_2_Ti_6_O_13_ and POMA (between 25 °C to 75 °C), it is reasonable to expect that ρ(T) of the ceramic-polymer composites displays a convolution of the two behaviors at ambient temperature and slightly above it (i.e., an increase in ρ(T) for the ceramic and a decrease for the polymer). However, given that the thermal expansion of the polymer exceeds by far the ceramic’s, it should further promote the separation of the ceramic particles, and improve percolation, therefore reducing the overall resistivity [33,34,35,36].

Considering the significant potential for fine-tuning the electrical behavior of Na_2_Ti_3_O_7_/Na_2_Ti_6_O_13_/POMA composites, a detailed study of the temperature dependence of their electrical properties is in order—a study that we carried out by means of complex impedance spectroscopy measurements.

## 2. Results and Discussion

### 2.1. X-ray Diffraction (XRD) Analysis

**Figure 1:**
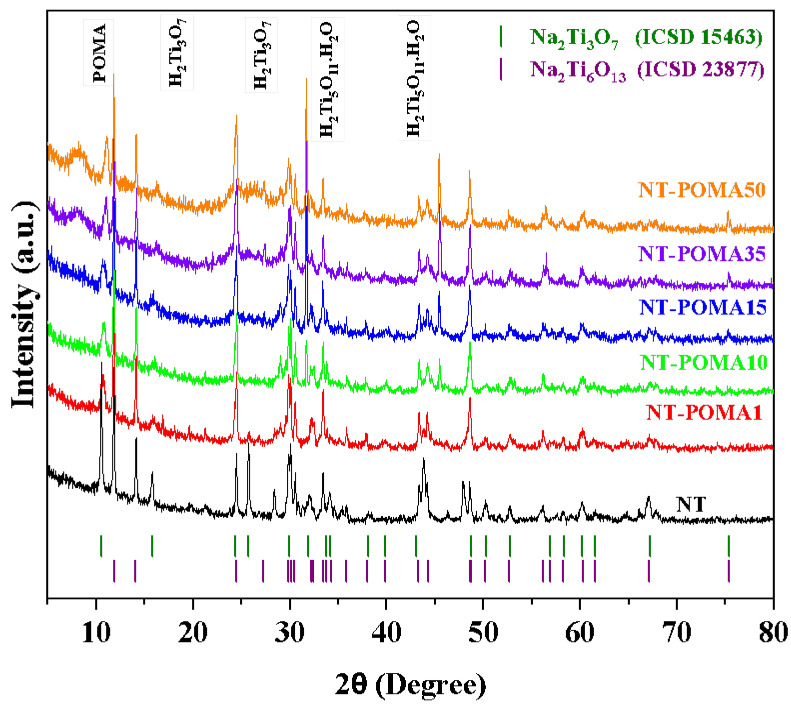
Diffraction patterns for NT, NT-POMA1, NT-POMA10, NT-POMA15, NT-POMA35 and NT-POMA50. The vertical lines indicate the Bragg peaks of Na_2_Ti_6_O_13_ and NaTi_3_O_7_ from the ICSD cards n° 23,877 and 15,463, respectively. The labels NT, NT-POMA1, NT-POMA10, NT-POMA15, NT-POMA35 and NT-POMA50 correspond to 0.0%, 0.1%, 10%, 15%, 35% and 50% of POMA content, respectively.

The XRD patterns of the Na_2_Ti_3_O_7_/Na_2_Ti_6_O_13_/POMA composites are shown in Figure 1. The intensity and sharpness of the reflections indicated a high degree of crystallinity. The characteristic crystallographic planes of the monoclinic structure (*P21m*) of sodium trititanate (Na_2_Ti_3_O_7_) were clearly identified according to the crystallographic information from the Inorganic Crystal Structure Database (ICSD). The crystalline lattice parameters were found to be *a* = 15.130 Å, *b* = 3.745 Å and *c* = 9.159 Å, with *α* = *γ* = 90° and *β* = 99.30°, in agreement with the ICSD card n°. 15,463 [37]. The high intensity diffraction peaks were identified at 2θ = 10.6°, 15.9° and 24.3° corresponding to the planes (200), (410) and (011), respectively. Sodium hexatitanate (Na_2_Ti_6_O_13_) has a space group *C2/m*; the lattice parameters and angles yielded by the structural refinement were *a* = 15.130 Å, *b* = 3.745 Å, *c* = 9.152 Å, *α* = *γ =* 90°, and *β =* 99.3°, respectively. All reflections (Figure 1) are in agreement with the crystallographic information in the ICSD card n°. 23,877, as well as in a previous report [38]. The crystallographic planes of greater intensity were indexed at 2θ = 11.8° (120), 14°, 24.4° (411), 29.8° (631) and 30° (720). 

The changes in the XRD pattern correlated well with the increase in the mass of POMA from NT-POMA1 to NT-POMA50, mainly in the 2θ regions centered at 8.2° and 26.6°. As the POMA mass was increased, the background raised slightly, leading to the formation of broad reflections at higher concentrations (which is typical of non-crystalline and semicrystalline materials), as found in organic matrices. The XRD analysis confirmed the formation of the Na_2_Ti_3_O_7_/Na_2_Ti_6_O_13_/POMA composites. However, given that the H^+^ ionic radius is slightly larger than Na^+^’s, the displacement of the (200) reflection from 2θ = 10.45° to 11.15° in NT-POMA10 and NT-POMA15 suggests a partial replacement of H^+^ for Na^+^, formingNa_2-x_H_x_Ti_3_O_7_. Other slight changes were also observed in 2θ = 15.68°, 29.09°, and 75.31°.

The profile of the peaks at 2è = 31.67° and 45.54° for NT-POMA35 and NT-POMA50 suggested the formation of H_2_Ti_5_O_11_.H_2_O (monoclinic-space group *C2/m*). Although it is possible to form the H_2_Ti_5_O_11_.H_2_O phase using traditional synthesis methods [39,40], to the best of our knowledge, this is the first time that the addition of POMA resulted in the phase transformation from Na_2_Ti_3_O_7_ to Na_2-x_H_x_Ti_3_O_7_.

### 2.2. Thermogravimetric (TGA) and Differential Thermal Analysis (DTA)

**Figure 2:**
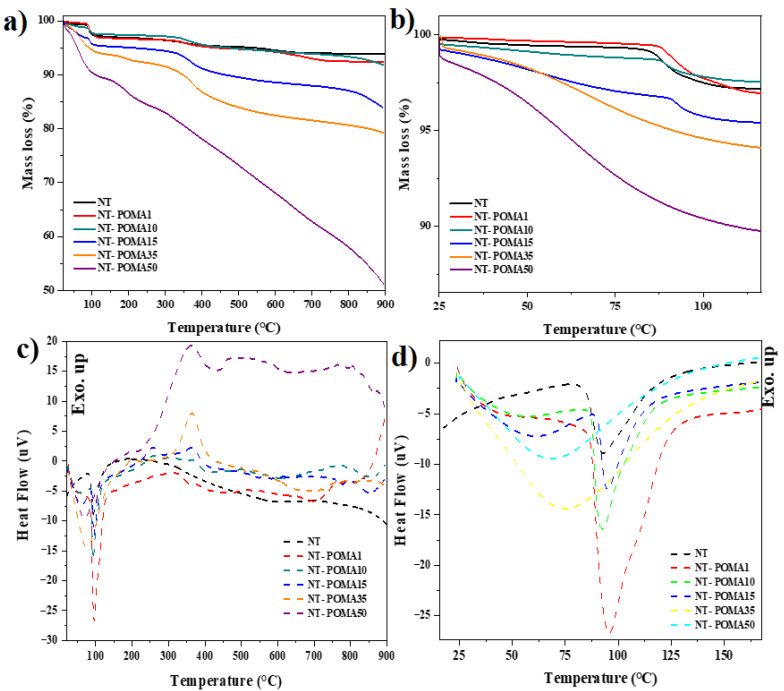
Thermogravimetric (TGA) and Differential Thermal Analysis (DTA) of Na_2_Ti_3_O_7_/Na_2_Ti_6_O_13_/POMA composites. (**a**,**b**) show the mass loss as a function of temperature determined from TGA; (**c**,**d**) show the heat flow as a function of temperature determined from DTA.

The TGA/DTA curves of the Na_2_Ti_3_O_7_/Na_2_Ti_6_O_13_/POMA composites, as well as the sodium titanate samples are shown in Figure 2. Three stages of mass loss, and one endothermic peak were observed in the TGA and DTA curves for the NT sample. The first thermal event between 25 °C and 100 °C was attributed to the evaporation of the water absorbed by the sample, corresponding to a mass loss of 2% and an endothermic peak around 93.2 °C. The second thermal event occurred in the range from 300 °C to 400 °C, with a mass variation of ~1%, attributed to the elimination of organic residues and hydroxyl groups (–OH) derived from the use of sodium hydroxide as a precursor material in the sonochemical synthesis [29,41,42]. According to previous XRD data, the NT sample is a 50% (*w*/*w*) mixture of the phases Na_2_Ti_3_O_7_ and Na_2_Ti_6_O_13_ [30]. Therefore, the last thermal event between 550 °C and 700 °C may be attributed to three processes: (i) the crystallization of Na_2_Ti_6_O_13_, which occurs near 550 °C [43,44]; (ii) a second process associated to the crystallization of the Na_2_Ti_3_O_7_ phase close to 680 °C [27,43,44], and finally (iii) a transformation to the more condensed and stable tunnel phase of Na_2_Ti_6_O_13_ through small atomic displacements in the open lamellar structure of Na_2_Ti_3_O_7_ above 700 °C [27,37,38,45].

The low variation of the NT total mass (~6%) and the absence of exothermic peaks associated with the possible processes from 550 °C to 700 °C are consistent with the scientific literature [27,29,41,43,44]. The NT sample was subjected to heat treatment at 900 °C to allow its recrystallization and identification by the XRD technique.

The highest mass reduction was ~49% for NT-POMA50, followed by ~21% for NT-POMA35, ~16% for NT-POMA15, and ~8% for NT-POMA10 and NT-POMA1. This result was expected because the sodium titanates have higher thermal stability than that of POMA, whose thermal degradation process occurs between 300 °C and 400 °C [35,46]. In general, the increase in POMA mass in the composites promoted the appearance of new endothermic and exothermic peaks in the DTA curves, as well as greater variation in the composites total mass in the TGA curves.

The TGA curves of NT-POMA1, NT-POMA10 and NT-POMA15 composites (Figure 2a) presented profiles similar to pure sodium titanate sample (NT), where three thermal events were identified. First, the mass loss between 25 °C and 120 °C was attributed to the loss of water (Figure 2b). Secondly, between 300 °C and 400 °C, the observed mass loss was due to the elimination of hydroxyl groups and the starting of the decomposition of the polymer chains. Finally, the crystallization and phase transformation processes of sodium titanates near 550 °C also resulted in a loss of mass.

The DTA curves from NT-POMA1 to NT-POMA15 composites showed endothermic peaks centered near 85–95 °C, while the peaks of NT-POMA35 and NT-POMA50 were broader, shallower, and centered near 60–70 °C, as shown in Figure 2d. This behavior is consistent with the release of water absorbed by the polymer [35,46,47,48]. Smaller exothermic peaks can be observed in the region between 200 °C and 400 °C. The first peak, centered near 260 °C, can be attributed to the degradation of the hydrochloric acid, which was used as a dopant in the synthesis of POMA, as well as to the degradation of lower molecular-weight fragments of the polymer chains. Peaks centered near 370 °C were suggestive of the onset of thermal degradation of the main polymer chains, as well as the elimination of hydroxyl groups from the backbone of the titanates [35,46,47,48].

The TGA curves of NT-POMA35 and NT-POMA50 composites presented three thermal events of mass loss below 500 °C. The first one was observed between 25 °C and 100 °C, which was accompanied by endothermic peaks around 70 °C. A second mass loss occurred from 100 °C to 230 °C, followed by small endothermic peaks around 200 °C. Finally, in the region between 230 °C and 450 °C, there was the third thermal event with endothermic peaks located close to 363 °C. The mass loss of NT-POMA50 composite above 500 °C became much more severe.

### 2.3. Electrical Behavior

**Figure 3:**
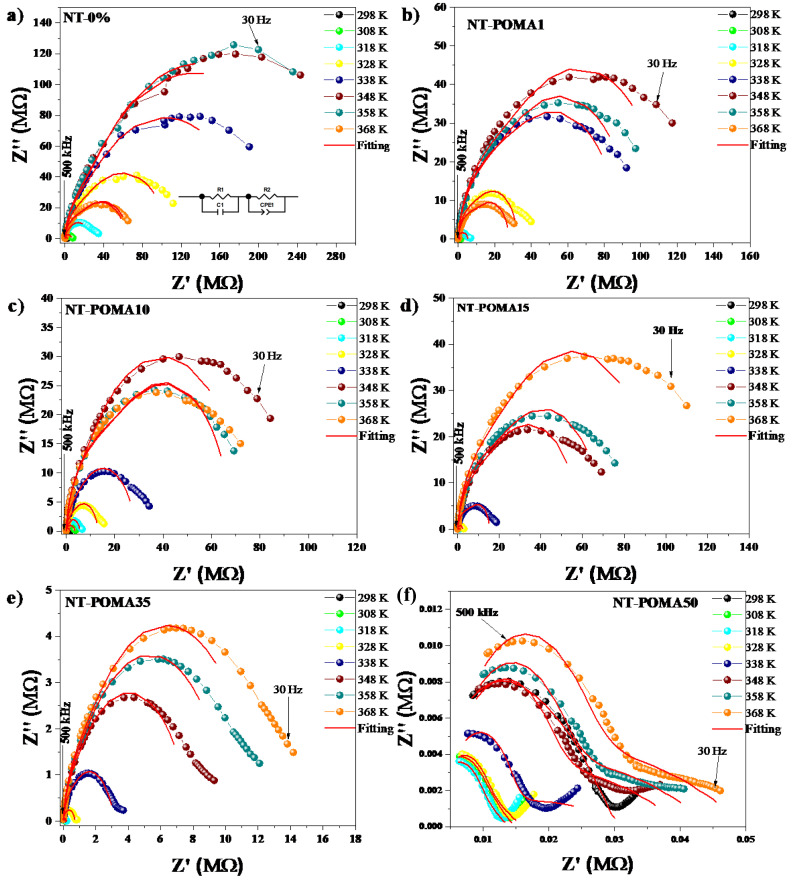
Isotherms of the Cole–Cole diagrams, with the behavior of the real (Z’) and imaginary (Z’’) part of the complex impedance for all the composites. The red lines correspond to fits using the equivalent circuit model. (**a**) NT, (**b**) NT-POMA1, (**c**) NT-POMA10, (**d**) NT-POMA15, (**e**) NT-POMA35 and (**f**) NT-POMA50 samples correspond to 0.0%, 0.1%, 10%, 15%, 35% and 50% of POMA content, respectively. The equivalent circuit used for the fit is shown in the inset of Figure 3a.

Figure 3 shows the Cole–Cole diagrams at different temperatures for all the Na_2_Ti_3_O_7_/Na_2_Ti_6_O_13_/POMA composites. This figure shows the behavior of the real (Z’) and imaginary (Z”) part of the complex impedance for different temperatures. Using the equivalent circuit model [49], the behavior of each sample and its main regions (grain and grain boundaries) can be determined through parameters such as resistance, capacitance, phase constant elements, etc. The diameter of semicircles are associated with the total resistance of the samples (Rt), which can be calculated from the equivalent circuit inserted in Figure 3a, where Rt = Rg + Rgb, and Rg(R1) and Rgb(R2) are grain and grain boundary resistances, respectively. 

The composites NT, NT-POMA1 and NT-POMA10 presented similar behaviors. The Cole–Cole semicircles diameter first increases with temperature up to 348 K, and then starts to decrease. From the point of view of electrical resistivity, this behavior can be seen as an initial increase in resistivity, followed by a decrease at higher temperatures. This increase in electrical resistivity with the temperature is unexpected in ceramics. In the case of the NT-POMA15 and NT-POMA35 composites, the diameter of the semicircles increased continuously as a function of temperature. This behavior can be attributed to the higher concentration of POMA, suggesting that in the composites of lower POMA concentration, the polymer is responsible for the drop in resistance at higher temperatures. It is reasonable to consider that an increase in POMA concentration in the ceramic matrix leads to the superposition of the conduction mechanisms of both materials. This would account for the decrease in the semicircles diameters in the Cole–Cole diagrams taken at the same temperature for all POMA concentrations. In the case of the NT-POMA50 composite, the behavior observed in the Cole–Cole diagram at different temperatures suggests that the conductivity of the polymer is becoming dominant. This suggests that for the higher concentrations of POMA, the decrease in the diameters of the Cole–Cole semicircles is controlled mainly by the POMA mass. As observed in Figure 3f, different from the other samples, now the semicircles do not close in the high frequency region. The behavior of this region is attributed to the response of the most homogeneous and semiconductor part of the ceramic material, that is, the grain.

**Figure 4:**
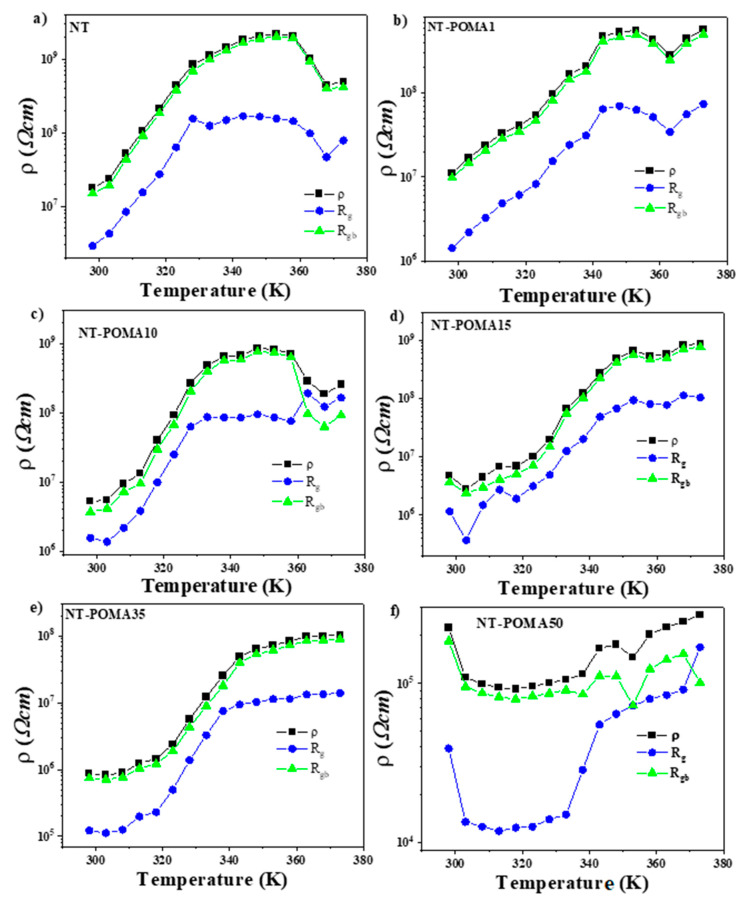
Temperature dependence of the total, grain and grain boundary resistivity for different compositions. (**a**) NT, (**b**) NT-POMA1, (**c**) NT-POMA10, (**d**) NT-POMA15, (**e**) NT-POMA35 and (**f**) NT-POMA50 samples correspond to 0.0%, 0.1%, 10%, 15%, 35% and 50% of POMA content, respectively.

The equivalent circuit model used to distinguish between the grain and grain boundaries is shown in the inset of Figure 3a. The resistivity values of the sample (*ρ*), grains (*ρ_g_*), and grain boundaries (*ρ_gb_*) for all samples were estimated from the fits of Z’ vs. Z” for each temperature considering the geometric factors for each sample. The behavior of *ρ*, *ρ_g_* and *ρ_gb_* as a function of temperature for the composites is shown in Figure 4. The temperature dependence of *ρ* and *ρ_gb_* were very close, clearly suggesting that the resistivity of these composites is governed by the grain boundaries. The behavior of *ρ_g_* versus T is quite similar for all composite compositions. The resistivity of the Na_2_Ti_3_O_7_/Na_2_Ti_6_O_13_/POMA composites from NT to POMA35 increased by nearly two orders of magnitude from 298K to 368K, suggesting that the resistivity is controlled mostly by the ceramic content. This behavior is known as positive temperature coefficient of resistivity (PTCR) and its origin is quite complex [33]. This temperature dependence is much reduced in the NT-POMA50 materials, suggesting that mainly the polymer is controlling the resistivity.

The PTCR effect in ceramics has been addressed effectively using a model proposed by Heywang and Jonker [34]. It considers the formation of a potential barrier at the grain boundaries region that can be written as:

(1) $$\Phi =\frac{e^{2}N_{s}{}^{2}}{8N_{d}\varepsilon_{0}\varepsilon_{gb}}$$

where *N_s_* is the concentration of trapped electrons, *N_d_* is the concentration of charge carriers, *Φ* is associated with the height of the potential barrier formed at the grain boundary, corresponding to the electron charge, and ε_0_ and ε_gb_ correspond to the permittivity values in vacuum and relative to the grain boundary, respectively. However, this model considers the ferroelectricity of barium titanate, and it is based on the existence of an iron-paraelectric phase transition that occurs in this material. Evidently, these considerations were not applied to the Na_2_Ti_3_O_7_/Na_2_Ti_6_O_13_/POMA composites, but otherwise the model is quite applicable.

An increase in resistivity of three orders of magnitude just above ambient temperature was recently reported for sodium titanate [31]. The authors correlated this anomaly to the combined effect of the disruption of the conductivity paths in the layers of the trititanate phase with the reduced conductivity of the tunnel channels of the hexatitanate phase upon an increase in temperature. In the latter, the sodium titanate samples used had 85/15 Na_2_Ti_6_O_13_/Na_2_Ti_3_O_7_ phase ratio. The electrical resistivity values obtained by the authors ranged from 10^5^–10^8^ Ω·cm. The difference in electrical resistivity values is possibly related to different compositions in the ceramic matrix, as well as the composite nature of the material used in this work, which contain different amounts of POMA.

#### Electrical Properties in the Time and Frequency Domains

The impedance spectrum as a function the frequency is a complex function:

(2) Z(ω,T)=Z′(ω,T)+iZ″(ω,T)=|Z(ω,T)|exp(iφ)

where *Z′(ω,T), Z″(ω,T)* are the real and imaginary component of *Z(ω,T),* respectively, Φ is the phase angle at the frequency ω and *T* is temperature [49]. In order to obtain the relaxation time, the impedance spectrum is written as:

(3) $$Z(\omega ,T)=Z_{\infty }[1-F(\omega ,T)]$$

where *Z*_∞_ is the real *Z* component at high frequency, and *F(ω,T)* represents the distribution function of the relaxation time that describes the relaxation type of the sample.

**Figure 5:**
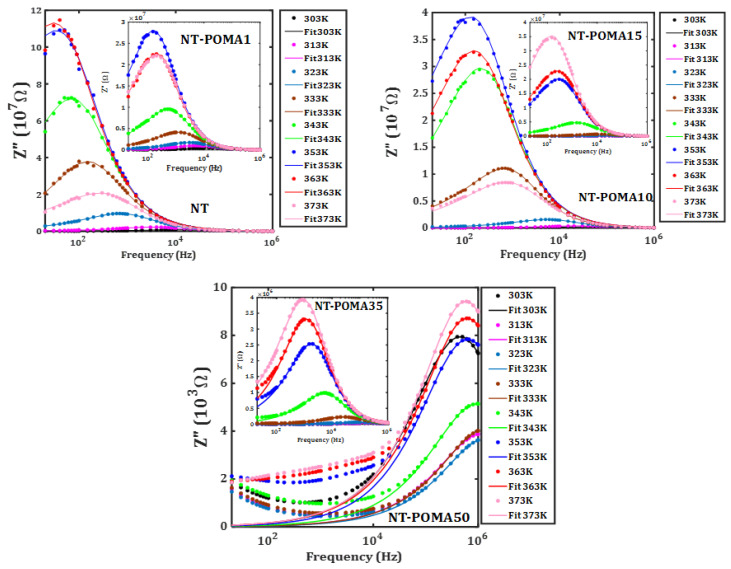
Frequency dependence of the imaginary component of the impedance from 303 K to 373 K. The samples NT, NT-POMA1, NT-POMA10, NT-POMA15, NT-POMA35 and NT-POMA50 correspond to 0.0%, 0.1%, 10%, 15%, 35% and 50% of POMA content, respectively.

Isotherms of the frequency dependence of the imaginary part of the impedance are shown in Figure 5 from 303 to 373 K. A peak in *Z*″ shifts to higher frequencies both with POMA concentration and temperature. These curves are fit to the best time relaxation distribution function. In all samples, the Havriliak-Negami distribution function:

(4) $$F_{HN}(\omega ,T)={\left[1+{\left(i\omega \tau \right)}^{\alpha }\right]}^{-\gamma }$$

describes the time relaxation distribution, (where τ is the relaxation time and α, γ are empirical parameters ranging between 0 and 1) [50,51,52]. In isotherms of *Z*″(f) for conducting materials, the magnitude of *Z*″ drops and the frequency for the maximum increases in temperature due to the decrease in the resistivity [53].

In time domain, the impedance is expressed as [50,51,52]:

(5) $$Z(\omega ,T)=Z_{\infty }\left[1-\int_{0}^{\infty }\left(-\frac{d\phi }{dt}\right)\exp(-i\omega t)dt\right]$$

where Φ is the relaxation function [50,51,52].

The relationship between the Havriliak–Negami distribution function *ρ*(*τ*) and *Φ* is expressed by:

(6) $$\phi (t)=\int_{0}^{\infty }\rho (\tau )\exp(-t/\tau )d\tau$$

Then, *Φ*(*t*) is fitted to the Kolhrausch–Williams–Watts (KWW) type function [49,50,51,52,54,55]:

(7) $$\phi (t)=\exp{\left(-t/\tau \right)}^{\beta }$$

where β is a one-dimensional parameter ranging from 0 to 1 and it is related to the correlated jumps of the charge carriers within its vicinity [51,52]. The average relaxation time (defined as the required time for the charge carriers to diffuse through the conduction path) is expressed as [51,52]:

(8) $$\langle \tau \rangle =\frac{\tau \cdot \Gamma \left(\frac{1}{\beta }\right)}{\beta }$$

where Γ is the Euler’s Gamma function.

**Figure 6:**
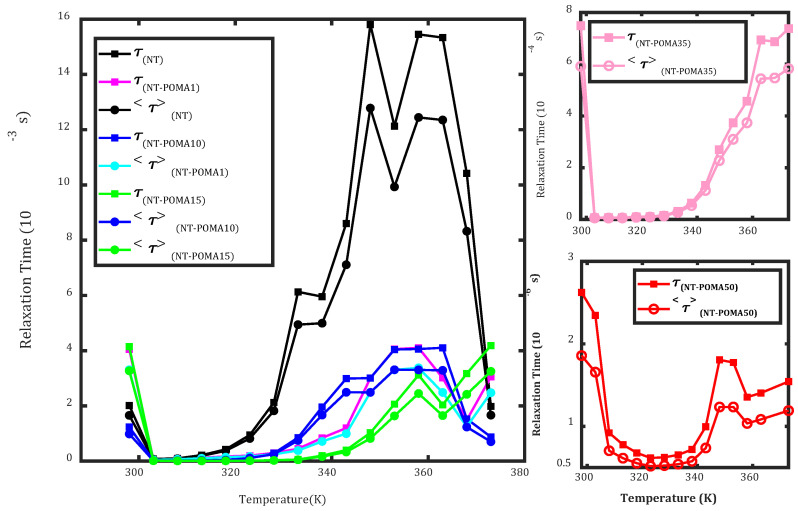
Temperature dependence of the average relaxation time <*τ*> and the relaxation time *τ* in the frequency domain for each sample. The data was obtained from the complex impedance formalism.

Figure 6 shows the dependence of the average relaxation time and the relaxation time with the temperature for each composite. Given the difficulty in obtaining an Arrhenius dependence of the relaxation time reliably, it is plausible that the PTCR effect was predominant in these composites.

## 3. Materials and Methods

The synthesis of Na_2_Ti_3_O_7_/Na_2_Ti_6_O_13_/POMA ceramic-polymer composites was carried out in several steps. First, Na_2_Ti_3_O_7_/Na_2_Ti_6_O_13_ and POMA were synthesized separately. Sodium titanate samples were produced by ultrasonic irradiation (487.5 W) in a 15 min cycle, using a Sonics Vibra-Cell VCX 750 sonicator, from a solution of titanium isopropoxide (C_12_H_28_O_4_Ti, Sigma-Aldrich, São Paulo, Brazil, 97%), diluted in isopropyl alcohol (C_3_H_7_OH, Synth, 99,5%), and a 1 mol·L^−1^ sodium hydroxide solution (NaOH, Synth, 98%), dissolved in deionized water. The solution obtained after sonication was dried in an oven at 110 °C for 12 h and heat treated at 900 °C for 1 h.

POMA was prepared by conventional chemical polymerization [35]. A solution of ammonium persulfate [(NH_4_)_2_S_2_O_8_, Sigma-Aldrich, São Paulo, Brazil, 98%] and 2 mol·L^−1^ hydrochloric acid (HCl, Synth, 37%) was slowly added to a mixture of o-anisidine (CH_3_OC_6_H_4_NH_2_, Sigma-Aldrich, São Paulo, Brazil, 99%) and 2 mol·L^−1^ HCl under constant stirring. The final solution remained under constant agitation for 3 h, followed by vacuum filtration with distilled water, and drying in a desiccator until a constant weight was reached.

The Na_2_Ti_3_O_7_/Na_2_Ti_6_O_13_/POMA composites were prepared in POMA mass concentrations of 0% (NT), 1% (NT-POMA1), 10% (NT-POMA10), 15% (NT-POMA15), 35% (NT-POMA35) and 50% (NT-POMA50), respectively. The polymer mass for each composition was added to 10 mL of distilled water for homogenization in an ultrasonic bath for 10 min. Subsequently, several amounts of powdered sodium titanate were added to the POMA suspensions. This step was necessary in order to achieve a physical mixture as homogeneous as possible. Each sample stayed in the ultrasonic bath for 10 min, followed by drying in an oven at 40 °C. 

The XRD patterns were obtained in a Panalytical Empyrean powder diffractometer, operating with copper monochromatic radiation (K_á_ = 1.5406 Å), 40 kV, 40 mA and diffraction angle 2è from 5° to 100°, corresponding to interplanar distances between ~1.0 and 17.5 Å. Rietveld refinements of the XRD patterns were carried out with Full Proof [36]. More details on synthesis methods and DRX refinements can be found elsewhere [31].

Thermal analysis was performed using a STA-7300 Hitachi instrument. All measurements of thermogravimetry (TGA) and differential thermal analysis (DTA) were collected simultaneously from ambient temperature to 900 °C, using a heating rate of 10 °C/min under a nitrogen gas flow of 100 mL/min. Approximately 10 mg of material was used for each measurement.

The electrical characterization of the samples was performed by means of a complex impedance spectroscopy analysis, using a Solartron 1260 Impedance Analyzer coupled to the furnace. The composite pellets for these measurements were 12 mm in diameter, 2 mm thick, and did not receive any heat treatment, in order to avoid polymer degradation. The measurements were performed from 298 K to 373 K in the frequency range of 10–1 MHz, using an excitation voltage of 500 mV corresponding to the applied electric field. 

## 4. Conclusions

The electrical characterization of Na_2_Ti_3_O_7_/Na_2_Ti_6_O_13_/POMA composites with different POMA contents was successfully carried out by means of measurements of the complex impedance. A positive temperature coefficient of resistivity was found in the samples of this study. The increase in electrical resistivity for the composites with POMA content up to 35% was about two orders of magnitude. For the composites with 50% of POMA, this effect was less visible. In all cases, the values of electrical resistivity were lower when compared to the grain boundary resistivity, which was responsible for this process. Similarly, an increase in relaxation time was observed with increasing temperature for all composites. In general, it was observed that the electrical properties of the studied composites varied, both with the polymer composition in the ceramic matrix and with temperature. These findings suggest that the Na_2_Ti_3_O_7_/Na_2_Ti_6_O_13_/POMA composites with up to 35% POMA can be considered as candidates for applications as thermistors.

## Data Availability

The data used to support the findings of this study are available from the corresponding authors upon request.
